# Supplementary material for: A unifying theory for top-heavy ecosystem structure in the ocean
Source: Nat Commun. 2018 Jan 2;9:23. doi: 10.1038/s41467-017-02450-y (PMC5750233; doi:10.1038/s41467-017-02450-y)
Supplement: Supplementary file 1 — Supplementary Information [file 41467_2017_2450_MOESM1_ESM.pdf]

**Supplementary Information**

Supplementary Table 1. Estimates of *PPMR* based on median prey size for large generalist predators (LGPs) and gigantic secondary consumers (GSCs).

| Predator | Prey | Predator Mass (kg) | Mean Prey Mass (kg) | Median Prey Mass (kg) | PPMR |
|---|---|---|---|---|---|
| **LGPs** | | | | | |
| Copper Shark | Varied | 100 | 0.46 | 0.08 | $1.25 \times 10^3$ |
| Bull Shark | Varied | 160 | 0.58 | 0.1 | $1.6 \times 10^3$ |
| Bluefin Tuna | Varied | 200 | 0.42 | 0.1 | $2 \times 10^3$ |
| **GSCs** | | | | | |
| Blue Whale | Krill | 100,000 | 0.004 | 0.004 | $2.5 \times 10^7$ |
| Whale Shark | Zooplankton | 20,000 | 0.001 | 0.001 | $2 \times 10^7$ |
| Manta Ray | Zooplankton | 1,000 | 0.001 | 0.001 | $1 \times 10^6$ |

Supplementary Table 2. Food web (or energy flow matrix) for size-structured food web (base case).

| Prey/Predator | Ind Mass (kg) | P | Z | SF | MF | LF | AP |
|---|---|---|---|---|---|---|---|
| Phytoplankton (P) | $10^{-8}$ | - | 1 | 0 | 0 | 0 | 0 |
| Zooplankton (Z) | $10^{-4}$ | - | - | 1 | 0 | 0 | 0 |
| Small Fish (SF) | $10^{-2}$ | - | - | - | 1 | 0 | 0 |
| Medium Fish (MF) | $10^{0}$ | - | - | - | - | 1 | 0 |
| Large Fish (LF) | $10^{2}$ | - | - | - | - | - | 1 |
| Apex Predator (AP) | $10^{4}$ | - | - | - | - | - | - |

Supplementary Table 3. Food web (or energy flow matrix) for size-structured food web with large generalist predators.

| **Prey/Predator** | **Ind Mass (kg)** | P | Z | SF | MF | LGP | AP |
|---|---|---|---|---|---|---|---|
| Phytoplankton (P) | $10^{-8}$ | - | 1 | 0 | 0 | 0 | 0 |
| Zooplankton (Z) | $10^{-4}$ | - | - | 1 | 0 | .1 | 0 |
| Small Fish (SF) | $10^{-2}$ | - | - | - | 1 | .3 | 0 |
| Medium Fish (MF) | $10^{0}$ | - | - | - | - | .6 | 0 |
| Large Generalist Predator (LGP) | $10^{2}$ | - | - | - | - | - | 1 |
| Apex Predator (AP) | $10^{4}$ | - | - | - | - | - | - |

Supplementary Table 4. Food web (or energy flow matrix) for size-structured food web with gigantic secondary consumers.

| Prey/Predator | Ind Mass (kg) | P | Z | SF | MF | LF | GSC |
|---|---|---|---|---|---|---|---|
| Phytoplankton (P) | $10^{-8}$ | - | 1 | 0 | 0 | 0 | 0 |
| Zooplankton (Z) | $10^{-4}$ | - | - | 1 | 0 | 0 | 1 |
| Small Fish (SF) | $10^{-2}$ | - | - | - | 1 | 0 | 0 |
| Medium Fish (MF) | $10^{0}$ | - | - | - | - | 1 | 0 |
| Large Fish (LF) | $10^{2}$ | - | - | - | - | - | 0 |
| Gigantic Secondary Consumer (GSC) | $10^{4}$ | - | - | - | - | - | - |

Supplementary Table 5. Food web (or energy flow matrix) for size-structured food web with large generalist predators and gigantic secondary consumers.

| **Prey/Predator** | **Ind Mass (kg)** | P | Z | SF | MF | LGP | GSC |
|---|---|---|---|---|---|---|---|
| Phytoplankton (P) | $10^{-8}$ | - | 1 | 0 | 0 | 0 | 0 |
| Zooplankton (Z) | $10^{-4}$ | - | - | 1 | 0 | .1 | 1 |
| Small Fish (SF) | $10^{-2}$ | - | - | - | 1 | .3 | 0 |
| Medium Fish (MF) | $10^{0}$ | - | - | - | - | .6 | 0 |
| Large Generalist Predator (LGP) | $10^{2}$ | - | - | - | - | - | 0 |
| Gigantic Secondary Consumer (GSC) | $10^{4}$ | - | - | - | - | - | - |

Supplementary Table 6. Food web (or energy flow matrix) for size-structured food web with large generalist predators and gigantic secondary consumers.

| **Prey/Predator** | **Ind Mass (kg)** | P | Z | SF | MF | LGP | GSC | AP |
|---|---|---|---|---|---|---|---|---|
| Phytoplankton (P) | $10^{-8}$ | - | 1 | 0 | 0 | 0 | 0 | - |
| Zooplankton (Z) | $10^{-4}$ | - | - | 1 | 0 | .1 | 1 | - |
| Small Fish (SF) | $10^{-2}$ | - | - | - | 1 | .3 | 0 | - |
| Medium Fish (MF) | $10^{0}$ | - | - | - | - | .6 | 0 | - |
| Large Generalist Predator (LGP) | $10^{2}$ | - | - | - | - | - | 0 | 0.5 |
| Gigantic Secondary Consumer (GSC) | $10^{4}$ | - | - | - | - | - | - | 0.5 |
| Apex Predators (AP) | $10^{2}$ | - | - | - | - | - | - | - |

Supplementary Table 7. Food web (or energy flow matrix) for simplified coral reef foodweb.

| **Prey/Predator** | **Ind Mass (kg)** | P | BA | Z | ZF | HF | PF | LGP |
|---|---|---|---|---|---|---|---|---|
| Phytoplankton (P) | $10^{-8}$ | - | - | .95 | 0 | .1 | 0 | 0 |
| Benthic Algae (BA) | $10^{-3}$ | - | - | .05 | .05 | .9 | 0 | 0 |
| Zooplankton (Z) | $10^{-4}$ | - | - | - | .95 | 0 | .1 | 0 |
| Planktivorous Fish (ZF) | $10^{-2}$ | - | - | - | - | - | .6 | .3 |
| Herbivorous Fish (HF) | $10^{-1}$ | - | - | - | - | - | .3 | .3 |
| Piscivorous Fish (PF) | $10^{0}$ | - | - | - | - | - | - | .4 |
| Lg. Gen. Pred. (LGP) | $10^{2}$ | - | - | - | - | - | - | - |

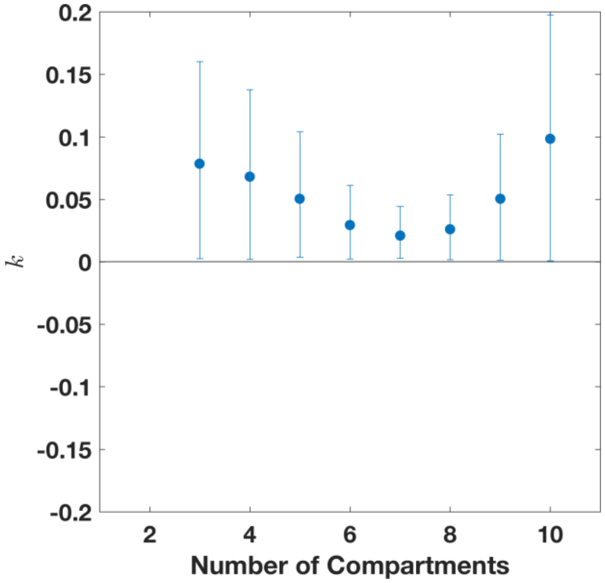


Supplementary Figure 1. Model sensitivity to number of trophic compartments with randomized food webs (5000 iterations) including LGPs and GSCs. The addition of LGPs and GSCs leads to positive biomass scaling and is largely insensitive to the number of trophic compartments with a mean of $k$ ~ 0.05. Error bars show 95% confidence intervals.

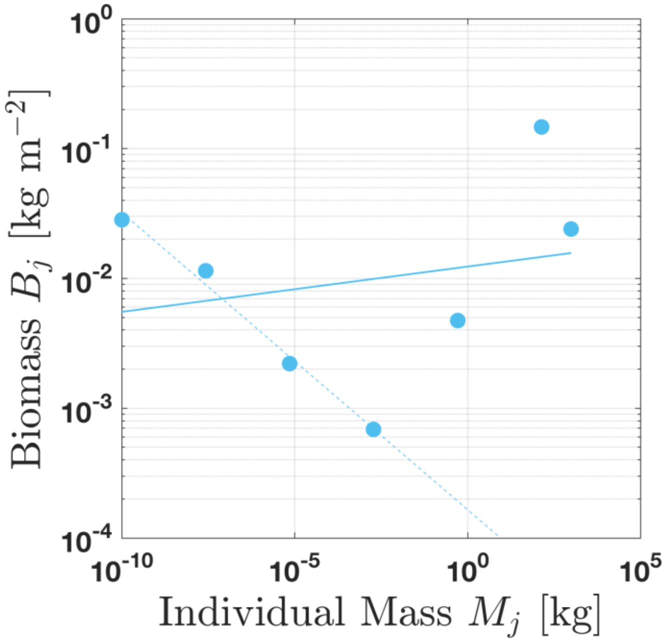


Supplementary Figure 2. Example of biomass distribution across trophic compartments for size-structured food web including LGPs, GSCs, and APs (Supplementary Table 6). Dashed line in each plot represents the slope of the biomass spectrum including only trophic compartments up to the fish size range (100 kg).

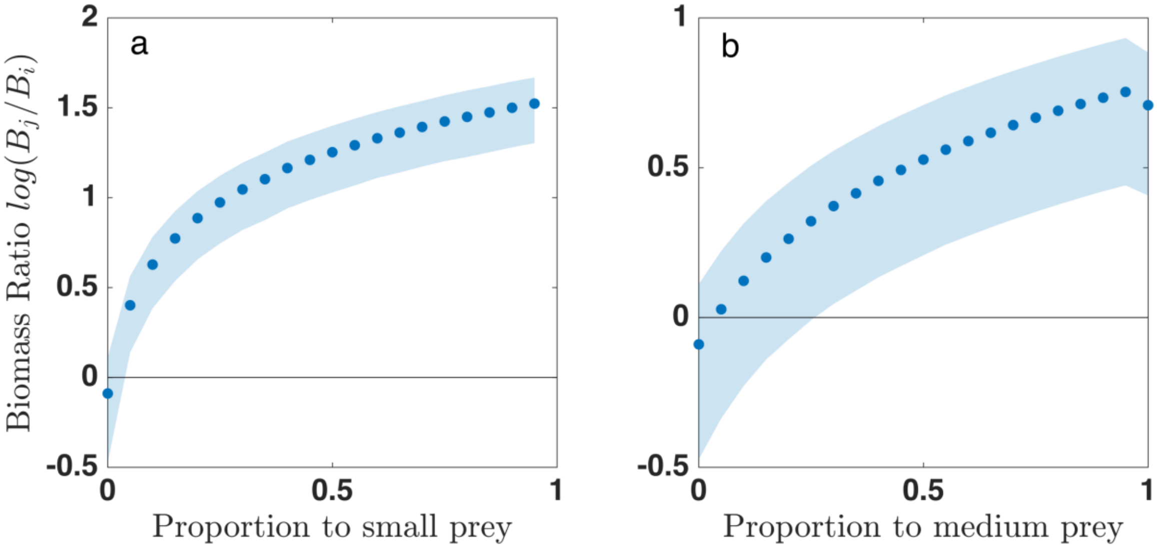


Supplementary Figure 3. Model sensitivity to diet matrices for LGPs. Prey proportion to small (a, zooplankton) and medium (b, small fish) prey items for LGPs. Biomass ratio > 0 indicates inverted trophic structure as the biomass of the predator will be greater than that of next smallest prey in size structured model. Shading indicates 95% confidence intervals.

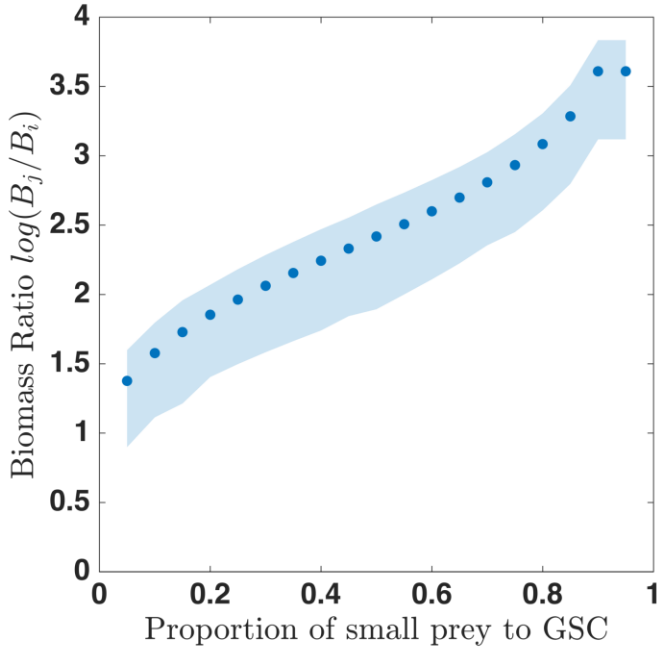


Supplementary Figure 4. Model sensitivity to proportion of energy in zooplankton class consumed by GSCs. A proportion of 1 indicates all 10% goes to GSCs. Biomass ratio > 0 indicates inverted trophic structure as the biomass of the predator will be greater than that of next smallest prey in size structured model. Shading indicates 95% confidence intervals.

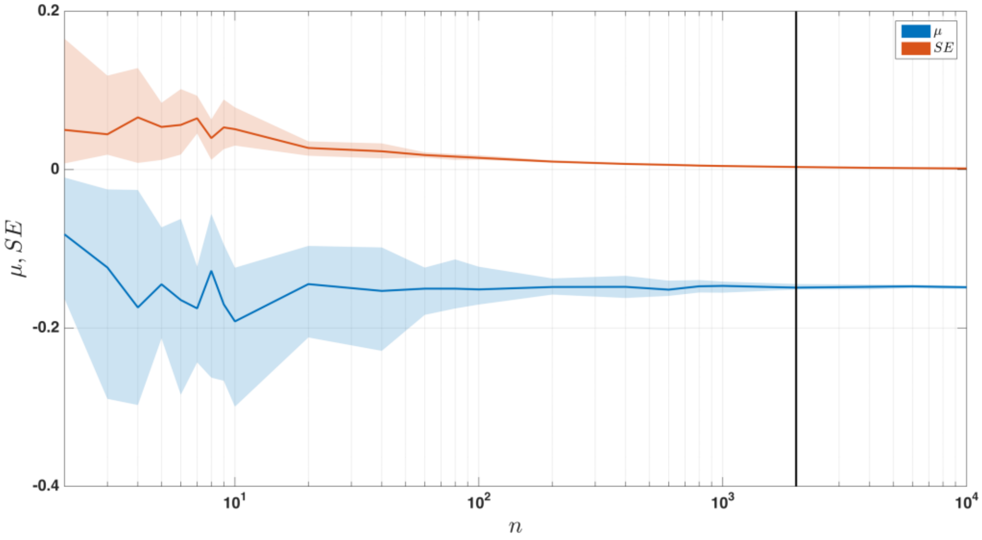


Supplementary Figure 5. Convergence of mean and standard error of *k* for randomly determined *TE*. Convergence occurs around 2000 samples. Shading indicates 95% confidence intervals.

Supplementary Note 1: Subroutine to compute ecosystem parameters. Full code to reproduce the model and figures available on github ().

```
#subfunction compute_sat_biomass that computes ecosystem parameters
def compute_sat_biomass(sst1,gpp1,par1,tweb,endo1,orgmass1,tclass1):

    #Constants based on Schramski et al 2015 (PNAS)
    k=8.62e-5 #Boltzman constant
    Po=2.16e9; #Prodcution Scaling Coefficient
    E=0.65; #Activation Energy
    beta=0.75; #Allometric Scaling (3/4 power)

    #trim file to number of trophic compartments
    nn=orgmass1.size
    flow2=np.zeros([nn,nn],dtype='f8')
    T=np.zeros(nn,dtype='f8')
    Pind=np.zeros(nn,dtype='f8')
    gross_prod=np.zeros(nn,dtype='f8')
    net_prod=np.zeros(nn,dtype='f8')
    population=np.zeros(nn,dtype='f8')
    group_massdensity=np.zeros(nn,dtype='f8')
    trop_trans=np.zeros(nn,dtype='f8')

    nper=0.5 #Fraction of gross production allocated to net production

    #compute region specific flow matrix with compartment specific TE
    for ii in range(0,nn):
        for jj in range(0,nn):
           flow2[ii,jj]=tweb[ii,jj]*gpp1

    #compute ecosystem properties ind production, gross production, net
    #production, population size, and group total biomass for each trophic
    #compartment based on Schramski et al 2015 (PNAS)

    for ii in range(0,nn):
        if endo1[ii]==0:
           T[ii]=273+sst1
        elif endo1[ii]==1:
           T[ii]=273+37
        Pind[ii]=Po*(orgmass1[ii]**beta)*np.exp(-E/(k*T[ii]))
        gross_prod[ii]=np.sum(flow2[:,ii])
        net_prod[ii]=nper*gross_prod[ii]
        population[ii]=net_prod[ii]/Pind[ii]
        group_massdensity[ii]=population[ii]*orgmass1[ii]

    return group_massdensity
```
